# Public Health Responses to COVID-19: Whose Lives Do We Flatten Along With “The Curve?”

**DOI:** 10.3389/fpubh.2020.564111

**Published:** 2020-12-02

**Authors:** Aravind Ganesh, Joao M. Rato, Venu M. Chennupati, Amanda Rojek, Anand Viswanathan

**Affiliations:** ^1^Department of Clinical Neurosciences, University of Calgary, Calgary, AB, Canada; ^2^Team Leader, Alberta COVID-19 Exposure Response Team (ACERT), Alberta Health Services, Calgary, AB, Canada; ^3^Chairman of Banco CTT, Lisbon, Portugal; ^4^Information Management School, Universidade Nova de Lisboa, Lisbon, Portugal; ^5^Chief Executive Officer, ZOLT Health Systems, Hyderabad, India; ^6^Fellow in Emerging Infectious Diseases, Emergency Department, Royal Melbourne Hospital, Melbourne, VIC, Australia; ^7^Centre for Integrated Critical Care, University of Melbourne, Melbourne, VIC, Australia; ^8^Director, Telestroke Services, Massachusetts General Hospital and Partners Healthcare, Boston, MA, United States; ^9^Harvard Medical School, Boston, MA, United States

**Keywords:** COVID 19, public health, socio-economic aspects, lockdown, social distancing

The Coronavirus 2019 (COVID-19) pandemic has received varying and evolving public health responses worldwide (1). Sweden remained largely open with health measures aimed most substantively at vulnerable groups, while South Korea implemented a large testing program, combined with extensive efforts to isolate infected people and trace/quarantine contacts. The United Kingdom (UK) considered various approaches before deciding on measures to isolate, quarantine, and promote social-distancing that were eased in mid-July (1); lockdown is now being re-implemented with a surging second wave (2). In contrast to early social-distancing measures in Canada to “flatten the curve,” American states adopted varying approaches, with many states having now relaxed their measures to differing extents (3). China adopted an aggressive approach of quarantining the affected Hubei province and isolating infected populations (4). India was under an ambitious 40-day lockdown, which was then extended until May-31 with districts designated as red/orange/green based on cumulative cases and doubling rate; red zones continued under full lockdown whereas orange/green zones had more relaxed measures (5). Gradual easing of restrictions (“unlock” 1.0 through 5.0) ensued, with lockdown measures nevertheless continuing in designated containment zones (6). Millions of people around the world still face public health measures of one form or another, raising the question: how stringent should government responses be in such pandemics (7), and how long can (or should) such measures continue?

## Arguments for Strict Measures

There are clear medical, socio-economic, and humanitarian arguments favoring strict, ongoing social-distancing or quarantine/lockdown measures, pending a resolution to the pandemic. Foremost, they may help rapidly halt COVID-19 spread (8). This can prevent healthcare systems from being overwhelmed, which can be catastrophic even in developed nations, as witnessed in Italy and Spain (9). In such “sharp curve” scenarios, acute/severe cases exceed hospital capacity in terms of equipment procurement, staffing, and bed number/acuity, with insufficient time to build further capacity. Healthcare providers can also be placed at unacceptable risk by community spread and dwindling personal protective equipment, further crippling the system and driving up deaths. Furthermore, groups like the elderly or those with disability become especially vulnerable to exclusionary practices, as utilitarian philosophies – predominant in such crises – unfortunately discriminate against these patients when allocating scarce resources like ventilators (10). The proportion in these categories will differ from country to country. For example, 6.2% of the Indian population is over 65-years of age vs. 22.8% in Italy; in contrast, 11.8% live with disability in higher-income countries vs. 18.0% in lower-/middle-income countries (LMICs) (11). A blanket lockdown may also circumvent the challenge of achieving completeness in case/contact isolation, posed in part by variable false-negative test results and the substantial prevalence of asymptomatic cases (12). A blanket lockdown can also be justified by criteria of economic efficiency, as infected individuals may not fully internalize the impact of their consumption/work decisions on viral transmission and may maintain unacceptable levels of economic interactions (13). This rationale is further supported if mortality becomes an increasing function of infections due to healthcare capacity issues (14). It may be economically optimal to tighten containment measures as the infection rate increases and relax them as it decreases.

In this regard, Sweden was an outlier in its decision to remain largely open, with closure of only high-schools/universities whilst advising isolation by symptomatic individuals and those over 70 (15). Unfortunately, Sweden experienced a higher mortality rate (about 559 per 1 million) than its Scandinavian neighbors and most other European nations except Italy, Spain, and the UK. Swedish intensive care unit (ICU) utilization rates remained lower than predicted, but this was correlated with more deaths in non-ICU patients, suggesting that patient prognosis may have driven ICU admissions, reducing healthcare load but at the cost of decreased survival in non-admitted patients (15).

## Arguments Against Aggressive or Prolonged Measures

On the other hand, there are equally compelling socio-economic and humanitarian arguments against aggressive/prolonged lockdown measures. Social-distancing is a tremendous economic privilege. To do so successfully, the person must have a home permitting isolation, whilst being able to obtain supplies without putting themselves/others at risk – this is not an option for slumdwellers or homeless individuals. The person should also be able to work from home or have back-up income. This is far from reality for daily-wage workers/laborers and small-business owners who suddenly find themselves without income, as was the case for roughly 434 million members of the Indian labor force during lockdown. Particularly in LMICs, governments may only be able to sustain economic freezing for a few months, especially if subsidizing wages for those unable to work or laid off. For example, the Center for Monitoring of Indian Economy (CMIE) projects that unemployment could spike to over 23%, with 50 million workers already estimated to lose their jobs (16). A survey by the non-profit organization Jan Sahas found that 42% of Indian migrant workers already ran out of rations half-way into the lockdown, with over 90% lacking any income (17). Consequently, many migrant workers were forced to return to their villages, creating further transmission risk. These challenges prompted the Indian government to intervene with a basic cash benefit of $40/month with some free food incentives for the unemployed. Even with such support, we may anticipate non-virus deaths among impoverished populations lacking resources to feed/shelter their families. This can be amplified by failure of preventative healthcare services, as seen with the Ebola outbreak in West Africa, during which deaths attributable to such failures exceeded those due to Ebola itself (18).

In addition, school closures may deprive socio-economically disadvantaged children of free meals, disrupt mental health, and place untenable child-care obligations on struggling families (19). Other unintended consequences include hospital avoidance by patients with emergent conditions (20) like heart disease or stroke, resulting in worse outcomes, and spikes in domestic violence as victims find themselves cooped up with their abusers (21). The very same seniors and people with disabilities whom we seek to protect from COVID-19 can end up worse off from loss of services and support networks (21). Furthermore, the longer the lockdown, the higher the number of companies that must close and the greater the loss of economic infrastructure. Workers who have accumulated valuable firm-specific skills will lose their jobs and part of their human capital is irrecoverable. This destruction of value is what governments are trying to avoid by providing support to firms to keep workers on their payroll during lockdown, and by facilitating credit to sustain treasuries of companies unable to produce (22). Of course, LMICs that cannot afford subsidizing firms will be unable to maintain part of their productive capabilities, hampering recovery prospects when this crisis abates.

## The Challenges of Finding a Balanced Approach

Seeking a compromise, some have advocated an intermediate approach, dubbed “the hammer and the dance,” where initial weeks of lockdown-style measures are followed by a period of relaxed measures allowing return-to-work for most healthy people, while presumably building healthcare capacity and increasing testing (23). However, with limited resources, a dramatic expansion of capacity may be infeasible for most nations in the short-term. Many lives are still doomed by “the hammer”; the most socio-economically deprived groups still will be devastated by weeks of lockdown unless there is extraordinary financial support, likely unaffordable for many LMICs. Furthermore, not everyone can “dance”: relaxation of distancing measures will likely generate a second peak within a few months, and medically vulnerable populations will again risk infection, but this time facing pressure to return to economic productivity.

Indeed, the *timing* and *duration* of public health interventions are just as critical as their stringency. Delays in applying these interventions and sub-optimal duration can limit their efficacy. One model suggested that extending measures by 1 month in Wuhan would delay a resurgence by two additional months; this may give systems a chance to recoup some resources and capacity (24). Another model examining India's initial 21-day lockdown strategy suggested that a 42–56 day lockdown would indeed be epidemiologically preferable (25). Yet such decisions are almost inevitable political minefields; epidemiological models indicate that the socially optimal lockdown length is always longer than the privately optimal length for individuals (26). The aforementioned Indian model also recognized a tremendous price to social and economic health with a longer lockdown (25).

In this regard, rather than a sustained uniform national lockdown, a region-specific approach based on geographic risk of spread may be a reasonable compromise. For example, the strictness of travel restrictions and associated penalties could be higher in areas with high population density (26). Others have advocated for a demographically-guided strategy, arguing that most gains of uniform policies may be realized by having stricter/longer lockdown policies for the oldest and/or most vulnerable groups alone. Such targeted policies, combined with measures to reduce between-group interactions, increased testing and isolation of infected individuals, appear to minimize economic losses and deaths in some models (27). Proponents of this approach (encapsulated in “the Great Barrington Declaration”) argue it will facilitate the development of “herd immunity” in the lower-risk population, eventually protecting vulnerable groups (28). However, as argued by proponents of the “John Snow Memorandum,” a separation of lower- and higher-risk groups is easier said than done, and uncontrolled transmission among younger people again risks substantial morbidity/mortality for the overall population (29). Furthermore, at present, there is little evidence for lasting protective immunity following COVID-19 infection.

Of course, it would be ideal if we could confidently differentiate between susceptible, infected, and recovered individuals, as we could fine-tune the intensity of economic interactions, consumption, and work, for these three groups. The first group would be more lightly contained, the second would be in lockdown, and the third would be making up for lost work as much as possible. To achieve a near-optimal situation, accurate testing becomes crucial for effective lockdown of infected individuals. However, testing standards vary worldwide, and different tests like RT-PCR (reverse transcriptase polymerase chain reaction) or rapid antigen tests have different false-positive/false-negative rates. Limited access to testing, lack of reporting infrastructure, and asymptomatic infections further complicate the picture, resulting in variable under-reporting of COVID-19 cases/deaths around the world and limiting the accuracy of testing/monitoring-based strategies (30). For instance, a study of testing data from 86 countries estimated cases and deaths as being 10.5 and 1.47 times official reports, respectively (31).

Reopening societies before adequate testing and contact tracing are in place can be catastrophic, as experienced by American states that led the pack in reopening their economies in early May. For example, Florida saw a 1,393% jump in daily cases since reopening and South Carolina experienced a 999% jump, with health officials estimating that they are still able to identify only about 14% of cases (32). On the other side of the world, its economy reeling from the prolonged nation-wide lockdown, and forcing a gradated approach to reopening, India is now reckoning with the challenges of a great rise in COVID-19 cases (currently second-highest in the world) despite still having similar healthcare capacity limitations as at the start of the pandemic (33). As policy-makers in India and other affected countries envision a life of greater economic reopening beyond the devastating summer/fall of 2020, they will no doubt seek to draw lessons from the experiences of their American counterparts about the perils involved.

## Conclusion

In the first instance, it can be easy to characterize either a full lockdown or relatively relaxed measures as being bold or decisive vs. callous or cold-hearted, but on closer examination it is evident that there are substantial proportions of the population that will be placed at risk in each case. Indeed, it can be exceptionally challenging to find strategies to address the pandemic that do not risk endangering one vulnerable population or another. Countries are therefore forced to play an unenviable optimization game of sorts to decide which group they are willing to “risk,” relatively speaking, and for how long, and what amount of economic consumption they are willing to trade off to avoid COVID-19-related deaths. As seen in the experiences of nations around the word, incentives for one approach or the other will be country-specific and driven by economic and demographic factors as well as prevailing cultural or political philosophies. Regardless of what approach is chosen in our race to “flatten the curve,” we must take into account the lives that we risk flattening with it, and do all we can to mitigate the damage.

## Author Contributions

The article was conceived through an inter-professional, international discussion among the authors who are clinician-scientists and economists. AG and AV are clinician-scientists with post-graduate training in epidemiology and population health, while AR has expertise in advancing clinical research during emerging infectious disease outbreaks, having done so during the Ebola outbreak. JR is a finance and public policy expert and was previously the chairman of the Portuguese Treasury and Public Debt Management Agency who worked with Portugal's debt restructuring after the 2008 financial crisis. VC is CEO of ZOLT health Technologies which runs one of the largest tele-health platforms for the Government of India, delivering medical care to millions of Indians in rural areas across the country. AG wrote the first draft and revised the paper. JR, VC, AR, and AV added conceptual insights, helped revise the paper, and approved the final manuscript. AG was the guarantor of the article. All authors contributed to the article and approved the submitted version.

## Patient and Public Involvement

JR and VC are non-medical authors. Patients and other members of the public were not involved in the creation of the article.

## Conflict of Interest

JR was employed by the company Banco CTT. VC was employed by the company ZOLT Health Systems. The remaining authors declare that the research was conducted in the absence of any commercial or financial relationships that could be construed as a potential conflict of interest.
